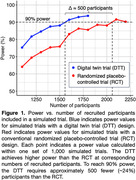# A digital twin technique using external observational data to reduce sample sizes in clinical trials on Alzheimer’s disease

**DOI:** 10.1002/alz.086975

**Published:** 2025-01-09

**Authors:** Daniel Andrews, D Louis Collins, the Alzheimer’s Disease Neuroimaging Initiative

**Affiliations:** ^1^ Department of Biomedical Engineering, McGill University, Montreal, QC Canada; ^2^ McConnell Brain Imaging Centre, Montreal Neurological Institute, McGill University, Montreal, QC Canada; ^3^ Department of Neurology and Neurosurgery, McGill University, Montreal, QC Canada

## Abstract

**Background:**

Randomized placebo‐controlled trials (RCTs) are the gold standard to evaluate efficacy of new drug treatments for Alzheimer’s disease. For example, the United States FDA approved the brain amyloid‐targeting drug lecanemab following CLARITY AD, Biogen and Eisai’s Phase 3 RCT. However, recruiting enough participants for a high‐powered and demographically representative trial is difficult and expensive. Fortunately, historical patient data from existing external observational studies of a disease can help populate RCTs [Thorlund et al. (2020). https://doi.org/10.2147/CLEP.S242097]. We propose a new trial framework that uses an external study to source “digital twins” for each trial participant. Using computer‐simulated trials mimicking CLARITY AD’s demographics and 18‐month duration, we show that our digital twin trial (DTT) has increased power compared to a conventional RCT.

**Method:**

A continuous time linear mixed model tracked CDRSB change‐from‐baseline (CDRSBΔbl) trajectories in 670 ADNI participants satisfying CLARITY AD inclusion criteria [clinicaltrials.gov/study/NCT03887455]. To simulate an RCT, we resampled and added noise to participants’ data, generating a desired sample size of “recruited” participants who we randomized 1:1 to “drug” and “placebo” groups. We calculated participants’ CDRSBΔbl scores at 18 months and simulated the drug effect as a 25% reduction in CDRSBΔbl. For each participant in our DTT, we used Gower’s distance on demographic and clinical baseline variables to identify 20 most‐similar real ADNI participants (the digital twins) from our original 670. Each original ADNI participant’s 18‐month CDRSBΔbl was calculated using the model. A z‐score was then calculated for each DTT participant’s 18‐month CDRSBΔbl relative to *their* digital twins. T‐tests were used to evaluate DTT drug vs. placebo group difference in mean z‐score and, separately, RCT group difference in mean 18‐month CDRSBΔbl. We simulated each trial 1,000 times. Power is the proportion of simulations with a statistically significant treatment group difference.

**Result:**

Figure 1 shows that 90% power is reached with approximately 500 fewer recruited participants in simulated DTTs (∼1,600 participants) compared to RCTs (∼2,100 participants).

**Conclusion:**

DTTs might require substantially fewer recruited participants to achieve the same power as conventional RCTs. This sample size reduction could facilitate recruitment for trials on Alzheimer’s and in rare diseases with low patient numbers.